# Quantitative measurements of inequality in geographic accessibility to pediatric care in Oita Prefecture, Japan: Standardization with complete spatial randomness

**DOI:** 10.1186/1472-6963-11-163

**Published:** 2011-07-07

**Authors:** Susumu Tanimura, Masayuki Shima

**Affiliations:** 1 Department of Public Health, Hyogo College of Medicine, 1-1 Mukogawa-cho, Nishinomiya-shi, Hyogo, Japan

## Abstract

**Background:**

A quantitative measurement of inequality in geographic accessibility to pediatric care as well as that of mean distance or travel time is very important for priority setting to ensure fair access to pediatric facilities. However, conventional techniques for measuring inequality is inappropriate in geographic settings. Since inequality measures of access distance or travel time is strongly influenced by the background geographic distribution patterns, they cannot be directly used for regional comparisons of geographic accessibility. The objective of this study is to resolve this issue by using a standardization approach.

**Methods:**

Travel times to the nearest pediatric care were calculated for all children in Oita Prefecture, Japan. Relative mean differences were considered as the inequality measure for secondary medical service areas, and were standardized with an expected value estimated from a Monte Carlo simulation based on complete spatial randomness.

**Results:**

The observed mean travel times in the area considered averaged 4.50 minutes, ranging from 1.83 to 7.02 minutes. The mean of the observed inequality measure was 1.1, ranging from 0.9 to 1.3. The expected values of the inequality measure varied according to the background geographic distribution pattern of children, which ranged from 0.3 to 0.7. After standardizing the observed inequality measure with the expected one, we found that the ranks of the inequality measure were reversed for the observed areas.

**Conclusions:**

Using the indicator proposed in this paper, it is possible to compare the inequality in geographic accessibility among regions. Such a comparison may facilitate priority setting in health policy and planning.

## Background

A scarcity of pediatricians has recently emerged as a major problem for child health care and welfare in Japan [1,2]. However, it should be noted that the total number of pediatricians nationwide has increased slightly in this decade [3]. This indicates that the shortage of pediatricians is more likely a result of unbalanced distribution, rather than a decrease in number [4].

As warnings of a potential collapse of the Japanese medical system for children has been given [3], it is critical to examine the geographic distribution of pediatricians and evaluate their accessibility.

A number of studies on geographic accessibility of health care have been published; some of them have presented sophisticated and complex techniques for measuring accessibility, making use of with higher-performance computational environments and advanced geographic information systems (GIS). For example, the floating catchment area method [5-8] is a GIS-based accessibility measures. Guagliardo [9] and Cromley and McLafferty [10] have reviewed the literature on geographic accessibility to health care. Although previous studies have proposed various innovative indicators, mean travel time to health care still remains a competent summary indicator when evaluating the distribution of health care providers, along with other classic indicators such as p-median and maximizing coverage. A distinguishing feature of mean travel time is that unlike complex indicators, it is based on simple concepts and can therefore be easily interpreted. Mean travel time in an area is calculated simply as the sum of each child's travel time between their home and the nearest pediatric facility, divided by the total child population in the area.

Mean travel time does not consider the distribution of travel time, i.e., whether the travel times are roughly equal amongst children or widely varying from the mean. A particular concern is that it can hide a number of people with considerably longer travel times than the average. Therefore, when describing geographic accessibility, not only the mean travel time but also the inequality indicator of travel time should be specified. This is analogous to specifying standard deviation along with a mean when summarizing a quantitative variable.

For measuring inequality in access to health care, researchers have proposed some indicators such as the Gini coefficient and Atkinson distributional measure [11,12]. These inequality measures are widely used by social scientists when reviewing economic inequality [13-15].

### Limitations of the inequality measure in geographic settings

**Figure 1:**
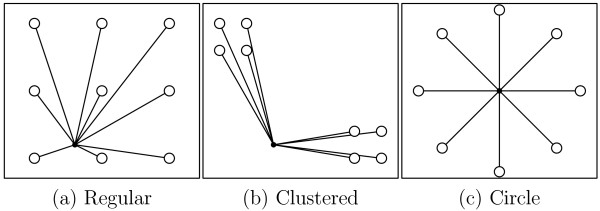
**Hypothetical examples of distribution patterns**. For theoretical consideration, children (white circles) and pediatric facilities (black bullets) are supposed to be distributed differently: (a) regular, (b) clustered, and (c) circular arrangements. Each line connects a child to a pediatric facility.

Unlike one-dimensional economic measures, our proposed inequality measure for travel time varies according to the two-dimensional distribution pattern of the location of children. Figure 1 shows hypothetical examples illustrating the limitations that the proposed method addresses. For simplicity, geographic accessibility is set as access distance (Euclidean distance) instead of travel time. In Figures 1(a) and 1(b), no ideal location for a pediatric facility is evident, meaning one that is equidistant from all children, irrespective of where the facility is allowed to be situated. An optimal location of a facility can be observed in Figure 1(c), that is, a central location with respect to all children. This implies that when we calculate the indicators of inequality in Figures 1(a),(b), and 1(c), we cannot directly compare their values because they have different connotations.

For priority setting in health policy and planning, it is extremely important to compare accessibility to pediatric facilities among many regions. For this purpose, we suggest that the indicator be standardized. The objective of this study is to develop a standardized method for measuring inequality in geographic accessibility to health care.

## Methods

### Study area

Oita Prefecture is located on Kyushu Island, in the southwestern part of Japan. It is almost entirely covered by mountains and has narrow coastal plains. Its population as of October 1, 2005, was 1.21 million, and its population density was 191 per square kilometer.

The prefecture was divided into six secondary medical service areas: Chubu, Hohi, Hokubu, Nambu, Seibu, and Tobu. In Japan, a secondary medical service area, which comprises one or more minor municipal districts (namely, cities, towns, or villages), is regarded as the basic unit for health care planning and administration.

### Data source

Patients aged 0-14 years are officially defined as pediatric patients. Therefore, we collected population data for children aged 0-14 years in Oita Prefecture, as of October 1, 2005, in the block level from the 2005 Population Census of Japan, reported by the Statistical Survey Department, Statistics Bureau, Japanese Ministry of Internal Affairs and Communications [16]. A block-level digital map of Oita Prefecture projected with Japan Plane Rectangular Coordinate System II was also obtained from the same data source.

A list of pediatric clinics and hospitals whose practicing pediatricians are certified by the Japan Pediatric Society was procured from the database service provided by the Oita Prefectural Government [17].

The road network data for the Oita Prefecture was derived from the digital national land information [18] released by the National and Regional Planning Bureau, Japanese Ministry of Land Infrastructure, Transport and Tourism.

### Measuring travel time

Although aggregation error is problematic if the zone area is too large [19,20], each block is small enough to allow the calculation of its centroid as the representative point of children residing in the block. We geocoded the address list of pediatric facilities to the same coordinate system of maps used in this study through the geocoding service provided by the Center for Spatial Information Science, University of Tokyo. The shortest path and its distance from the centroid to the nearest pediatric facility was calculated in kilometers. The nearest pediatric facility was determined solely by distance, regardless of whether the selected facility was inside or outside the targeted secondary medical care service area. Following the legal speed limits, the speed of traveling with car is assumed with a road classification as followed: 80 km/h for a toll road, 60 km/h for a national road or principal local road, and 40 km/h for others. A travel time was estimated from the network distance and the speed along each road segment in the route.

In Oita Prefecture, a mother and her child would usually travel to the nearest pediatric facility in the family car. If the facility is very close to their residence, the mother will travel on foot with her child, since bicycles are not very popular in this area and the public buses are not a very practical option in this case. Therefore, multiple modes of transport are not accounted for and car travel times are used for all individuals. While this will underestimate travel times for those on public transport or walking, for the reasons explained above we expect the number of people in this category to be small.

### Definition of indicators

For measuring statistical dispersion in this study, we use the relative mean difference *D*. This is the mean of the pairwise differences divided by the sample, or

Here *n *denotes the population size, *x_i _*(*i *= 1, 2, ..., *n*) is the travel time between the *i*th child and the nearest pediatric facility, *x_j _*is the corresponding travel time for the *j*th child, and  is the sample mean, which is always finite and nonzero.

*D *is mathematically equal to twice the value of Gini coefficient [21], which is widely used for inequality research in development economics and other fields [22,23].

### Monte Carlo simulation

Unlike one-dimensional variables such as income and poverty, the variable *D *of the travel time should be standardized because the influence of background geographic distribution pattern of children cannot be ignored.

For this standardization, we use Monte Carlo simulation to estimate , which is the expected value of *D *when the child locations are fixed and the pediatric facilities are uniformly and independently distributed throughout the study region. For each simulation, the pediatric facilities are randomly relocated in the area, using complete spatial randomness (CSR). CSR, or more formally a homogeneous Poisson spatial point process, has the following conditions: (1) *N*(*A*), the number of point events in region *A*, follows a Poisson distribution with mean of *λ*|*A*|; (2) given *N*(*A*), events in region *A *occurs following uniform distribution of *A*, where |*A*| denotes the area of region *A*, and *λ *is the density of points within the defined area [24,25].

When evaluating the inequality in travel time to pediatric care, the both distribution patterns of pediatric facilities and children are involved. Using CSR for relocating pediatric facilities cancels the effect of the distribution pattern of pediatric facilities (i.e., spatial autocorrelation) because the hypothesis of CSR assumes that the locations of these points are independent of each other. Therefore, after the CSR was applied in the simulation, only the effect of child distribution remained in the inequality measure, which is standardized in this study as follows.

The simulation steps for the target region (the secondary medical care service area) are as follows:

1. The pediatric facilities were divided into two groups: outside and inside the target region;

2. The pediatric facilities inside the target region were relocated using the CSR, while the remaining pediatric facilities were kept in their original locations;

3. *D *was calculated for children within the target area, where children were allowed access to the nearest pediatric facility regardless of whether the selected facility was inside or outside the target region;

4. These steps were repeated 99 times;

5. After 99 repetitions, 99 *D*s and 1 observed case (a total of 100) were combined, and the mean was computed as .

The standardized ratio is defined as , where *D *is the relative mean difference of the observed travel time and  is the expected value of *D*. In this study,  was used as a new indicator to evaluate the regional difference in the inequality of geographic accessibility to pediatric facilities.

The Oita Prefecture has six secondary medical service areas, and these simulation steps were applied independently in each area.

All calculations and simulations were performed by using R version 2.9.0 [26]. We used the **spgrass6 **[27] package and GRASS GIS [28] version 6.5 for measuring travel time, and the **splancs **package [29] for the simulating CSR.

## Results

### Distribution of children and pediatric facilities

**Figure 2:**
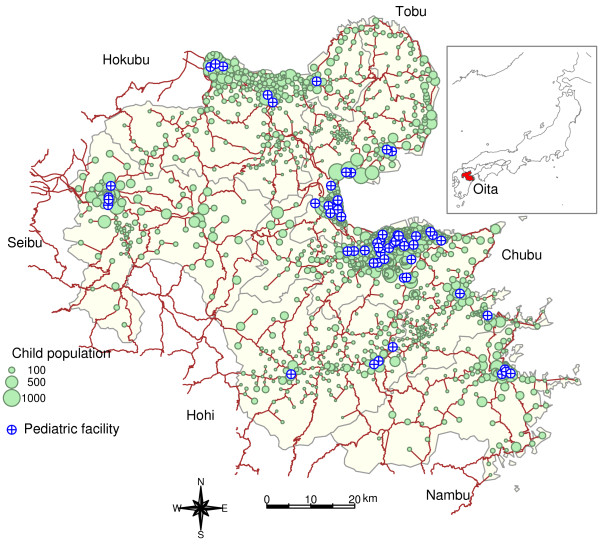
**Map of children and pediatric care facilities in Oita Prefecture**. The distribution of child population is shown using a green proportional symbol, the area of which corresponds to the population. The location of the pediatric facilities is denoted as in blue. Brown and gray solid lines are used to denote the road network and the administrative boundaries, respectively. The small top-right window shows a map of Japan with the shaded region representing Oita Prefecture.

Figure 2 shows the distribution of children and pediatric facilities, road network, and administrative boundaries of the secondary medical care area. A large number of children reside in the coastal areas of the prefecture, with the exception of the Seibu and Hohi areas. Most of the locations of the facilities corresponded to the population clusters of children. The number of pediatric facilities also corresponded to the size of children clusters. The child population per pediatric facility ranged from 1918.2 to 3865.0, whereas child population and the number of pediatric facilities varied greatly (Table 1).

**Table 1:** Children and pediatric providers by secondary medical care service area

Area	Child population	Number of Pediatric facilities	Child population per pediatric facility	Geographic area (km^2^)
Tobu	26855	14	1918.2	803.03
Chubu	80926	30	2697.5	1190.98
Nambu	10203	4	2550.8	903.40
Hohi	7386	4	1846.5	1081.03
Seibu	14416	4	3604.0	1224.04
Hokubu	23190	6	3865.0	1136.85

Total	162976	62	2628.6	6339.33

### Distance and *D*

**Figure 3:**
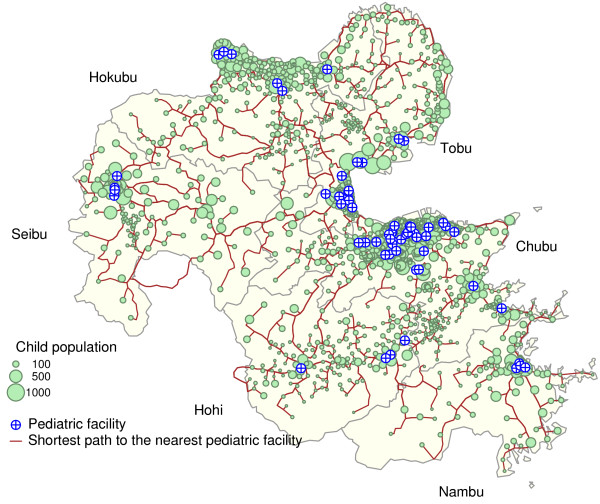
**Shortest path from a child to the nearest pediatric facility**. The shortest path from a child to the nearest pediatric facility is drawn with a brown solid line. The remaining symbols and lines are the same as in Figure 2.

The shortest path between a child's residence and the nearest pediatric facility over the road network is illustrated in Figure 3. Note that Figures 2 and 3 show similar features, yet the brown lines in Figure 2 denote the road network itself whereas the those in Figure 3 denote the shortest path on the road network. The two figures show that the shortest paths cover almost the entire road network, except the western peripheral zone (e.g., the western side of Nambu area). The travel time is summarized by area in Table 2. The mean of the observed mean travel times in the observed areas was 4.492 minutes, which is longer than the mean distance for the whole study area (Table 2). The Chubu area had the shortest mean travel time, whereas the Seibu area had the longest time, that is, nearly 4 times longer than that in Chubu. The mean of the observed *D *by area was 1.11, ranging from 0.93 to 1.34, whereas *D *for the entire study region was 1.24. The ranks of the areas according to the value of *D*, which is not yet standardized, in ascending order are shown in Table 2. The best (the least inequality) and the worst (the greatest inequality) area were Hohi and Tobu, respectively.

**Table 2:** Mean travel time and *D *for the shortest path to the nearest pediatric facility

Area	Travel time (min)	*D*	Rank
Chubu	1.830	1.081	3
Hohi	5.994	0.930	1
Hokubu	3.466	1.077	2
Nambu	4.639	1.147	5
Seibu	7.023	1.083	4
Tobu	4.002	1.337	6

Whole area	3.245	1.239	

*D *is the inequality measure that is not standardized. The rank of *D *is shown in ascending order (the higher the rank, the more equal is the access).

**Figure 4:**
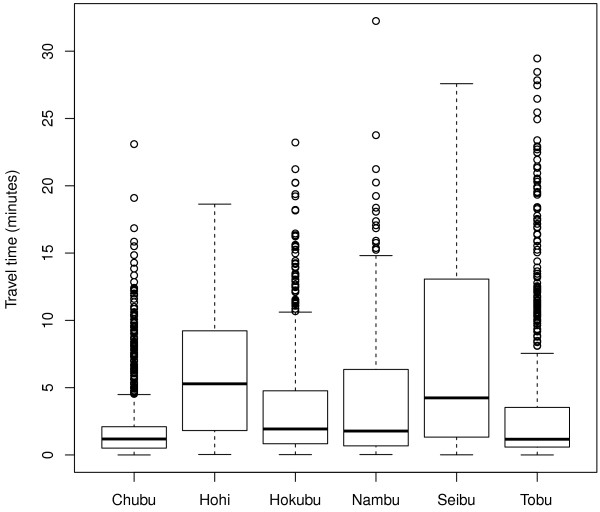
**Box plot of the travel time by secondary medical service area**. The travel time (in minutes) of the shortest path by secondary medical service area is summarized. The box denotes the interquartile range, and the centerline of the box expresses the median. The extreme data points are shown when there exist outside whiskers that are 1.5 times longer than the interquartile range.

A box plot of the travel time for each secondary medical service area depicted the distribution of the travel time in each area (Figure 4). The medians were smaller in the Tobu (1.17 minutes) and Chubu areas (1.19 kilometers) than those in other areas: Hohi (5.29 minutes), Hokubu (1.93 minutes), Nambu (1.78 minutes), and Seibu (4.24 minutes). The lower and upper quartiles (1.33-13.07 minutes) were larger in the Seibu area than those of other areas: Chubu (0.51-2.10 minutes), Hohi (1.81-9.23 minutes), Hokubu (0.83-4.77 minutes), Nambu (0.68-6.35 minutes), and Tobu (0.59-3.53 minutes). In the Chubu, Hokubu, and Tobu areas, we observed many outliers that deviated toward the longer distance side.

### Simulation with CSR

On the basis of the hypothetical example shown in Figure 1, we also computed  using the same approach as in our real observed data except using access Euclidean distance instead of travel time, to probe the background effect under different distribution patterns in the theoretical examples.  had substantially different values: (a) 1.776, (b) 1.804, and (c) 1.676.

In Oita Prefecture,  varied across areas, ranging from 0.26 to 0.73 (Table 3). All  had considerably smaller values than the corresponding *D*s (Table 2); further, the range of the variation in  was larger than that in *D*.

**Table 3:** Summary of the Monte Carlo simulation by area

			Rank
Chubu	0.456	2.370	2
Hohi	0.340	2.736	3
Hokubu	0.341	3.159	5
Nambu	0.257	4.464	6
Seibu	0.367	2.950	4
Tobu	0.732	1.827	1

Mean	0.415	2.92	

*D *for the travel time to the nearest pediatric facility was repeatedly calculated using a Monte Carlo simulation by area.  is the expected value of the observed *D *(i.e., the mean of *D *from one observation and 99 simulations).  is a standardized in-equality measure proposed in this paper as a new indicator. The rank of  is shown in ascending order (the higher the rank, the more equal is the access).

Addressing the main objective of this study, we present the standardized ratio, , for each area in Table 3. The largest and smallest standardized ratios were observed in the Tobu and Nambu areas, respectively. Tables 2 and 3 show that the rank of Tobu area changed dramatically from the last to the first, while the rank of the Nambu area decreased by one and became to the last. The Seibu area had the same rank. The rank of other areas decreased by one or two (Tables 2 and 3).

## Discussion

In this study, the inequality measure was standardized using an expected value that was estimated by CSR and a Monte Carlo simulation. The expected value reflects the background geographic distribution of the location of children and also the layout of the road network in each area. Thus, all background factors influencing to measuring travel time were integrated into the expected value. This made it possible to adjust for regional differences in child distribution, and to compare the inequality in geographic access to pediatric facilities across various regions in Oita Prefecture, Japan, in a standardized framework.

The reversal in inequality ranking after standardization (Tables 2 and 3) indicated that the adjusted inequality indicator can reveal the actual situation regarding the inequality in travel time to pediatric care. Further, it showed that the use of an unadjusted inequality indicator can lead to incorrect conclusions for priority setting in health resource allocation.

The fact that the  in theoretical examples (Figure 1) showed different values supported our hypothesis that the inequality measure varied considerably with the background geographic distribution pattern. Estimating  was a key issue in this study. For the simulation, we proposed the use of CSR for relocating the pediatric facilities. While determining the spatially random location for pediatric facilities, we set the boundary of the relocated area as within the secondary medical service area. Although we did not examine the effect of relocating the pediatric facilities outside the boundary, the effect of standardization in our results is meaningful because planning and decision making with regard to health services is undertaken at the secondary medical service area level.

We chose Oita Prefecture as the study area because it is a typical, or ordinary, prefecture that is located outside any metropolitan area in Japan. While it does have some urban areas that are densely populated, it is mainly dominated by villages and mountainous areas. Oita Prefecture has common demographic indicators and an ordinary number of pediatricians and facilities per children (see the methods section and Table 1; data regarding other prefectures is not provided). Therefore, the results in this study may be more generalizable than those that could be obtained from other prefectures in Japan, aside from metropolitan areas such as Tokyo and Osaka.

In this study, geographic accessibility was simply defined using the nearest pediatric care facility. Although the proposed indicators in other studies [30,5,19,33] integrated some or all of the facilities, we did not employ this approach because the pediatric facilities situated at a distance from the nearest facility were considered to be very rarely visited. Also, a more complex approach would not have the simple explanation and interpretation the method in this paper describes.

This study assumed that a child patient would be taken to the pediatric facilities closest to his/her residence. This assumption is not always true because of the "activity space" involved in the daily travel patterns of individuals [11]. For example, a mother may take her child to a pediatric facility that is close to her working place or to the child's nursery school. She might also take her child to a well-known pediatric facility or a facility where a friend is employed, even if the facility may not be located near her residence. Since such cases can distort the result, further investigation is required to overcome this limitation.

With the recent development of GIS, several other types of distance measures are being used in health service studies, including road distance [34-36], travel time [37-40], and Minkowski distance [41]. Since travel times provide a better indication of geographic barriers to pediatric care services than road network distance, the travel time was adopted in this study for measuring geographic accessibility. Yet, the calculation of travel time in this study had an intrinsic problem in setting more realistic travel speed (e.g., speed in flat area or on a steep and winding road). The legal speed limits, we used in this study, are defined by Japanese Road Structure Ordinance and enforced by the police. Since the ordinance takes account for traffic volume, road condition, and geography (i.e., categorized with flat or mountainous area), the minimum requirement for realistic travel time may be satisfied. For overcoming the limitations, further investigation with actual observation of the average speed is needed to ensure more realistic estimation. With regard to the computational cost, on the high-end personal computer used in this study, the Euclidean distance calculation for each iteration took less than a second, while calculations of travel time took almost 40 minutes. Accordingly, for a large data set and/or many iterations, the trade-off between process time and accuracy should be considered carefully. Nevertheless, our approach is theoretically applicable to all other types of distance.

Other studies have examined the effect of different modes of transportation (e.g., [42,43]). Since the dominant mode is an automobile as mentioned in methods section except the case that the pediatric facility is very close to the residence, we assumed that automobiles are the only mode of transport for the sake of simplicity. This could be a limitation in this study. For instance, where the network distance from residence to the nearest pediatric facility is only 100 meters along with principal regional road (60 km/h), the travel time is computed as 0.1 minutes (6 seconds). Nevertheless, we kept the assumption of travel mode because the threshold between automobile travel and walking is unclear. In further studies, we may adopt a cutoff value of network distance for conditional calculation after some additional observational studies.

In this study, we focused on the variations in proximity and did not consider personal, organizational, and financial barriers. Evidently, geographic accessibility is only one factor affecting accessibility to health services [11]; however, the physical barriers involved in geographic accessibility tend to be particularly important in areas with limited health services. Furthermore, some studies suggest that poor geographic accessibility reduces the use of health care services [44,37,46], leading to poorer health outcomes [44,47,48].

As mentioned in the background, various inequality measures of income distribution, apart from relative mean difference (Gini coefficient), have been proposed by many economists: the decimal ratio, Robin Hood index [49], Atkinson index [50], and Theil's entropy measure [51]. These measures can be applied in our approach simply by replacing the *D *with the others. However, we would expect the results to be similar because previous studies have reported very high correlations among these measures [52].

The methodology presented here has a number of limitations and simplifying assumptions, and the studies of geographic accessibility to health care may have only limited impact on the alleviation of inequalities in this health. Nevertheless, we believe that these findings are meaningful and the objectives of this type of research are of value to society.

## Conclusions

In this paper, we successfully demonstrated the standardization of *D *with an expected value under CSR. We concluded that adjusting the background geographic distribution pattern makes it possible to examine the regional differences in the inequality in geographic accessibility to health care. Furthermore, a comparison of regions by considering the mean travel time and the proposed indicator may assist in setting of priorities for ensuring fair access to pediatric facilities.

## Competing interests

The authors declare that they have no competing interests.

## Authors' contributions

The contributions of each author in this study are specified as follows: ST conceived the study, participated in its design and coordination, and performed the statistical analysis. MS participated in its design and coordination. Both the authors have read, revised, and approved the final manuscript and have agreed to its submission for publication.

## Pre-publication history

The pre-publication history for this paper can be accessed here:

http://www.biomedcentral.com/1472-6963/11/163/prepub
